# Long-Lasting Cognitive and Physical Impairment After Recreational Use of the Semisynthetic Cannabinoid Hexahydrocannabinonyl (HHC-C9): A Case Report

**DOI:** 10.3390/reports8030176

**Published:** 2025-09-11

**Authors:** Nanna Reiter, Dorte Fris Palmqvist, Gro Borges Larsen, Mathilde Emilie Høi, Brian Schou Rasmussen, Ragnar Thomsen

**Affiliations:** 1 Department of Anaesthesia and Intensive Care, Copenhagen University Hospital—Bispebjerg and Frederiksberg, 2400 Copenhagen, Denmark; 2 Department of Internal Medicine, Kolding Hospital, 6000 Kolding, Denmark; 3 Department of Neurology, Kolding Hospital, 6000 Kolding, Denmark; mathilde.emilie.hoi2@rsyd.dk; 4 Section of Forensic Chemistry, Department of Forensic Medicine, Faculty of Health and Medical Sciences, University of Copenhagen, 2100 Copenhagen, Denmark

**Keywords:** hexahydrocannabinol, HHC-C9, CC9, semisynthetic cannabinoid, sedation

## Abstract

**Background and Clinical Significance**: The recreational use of semisynthetic cannabinoids (SSCs) is increasing, and SSCs account for more than 40% of all new substances reported at the European level. Although designed to mimic the effects of tetrahydrocannabinol (THC), the primary psychoactive compound in cannabis, evidence suggests that certain SSCs may elicit stronger, prolonged and unintended pharmacological effects. SSCs are easily accessible, particularly via online retailers, but in some countries, SSCs are also sold in convenience stores or specialty stores selling legal low-THC or cannabidiol (CBD) products. Often, SSCs are sold as “legal highs” and are found in various forms, including herbal mixtures (spice), vape products, and edibles such as cookies and candies, specifically targeting young users, including children. The products are frequently mislabeled and sold as souvenirs or aromatic potpourri to bypass regulations. **Case Presentation**: We present a case of a male in his early forties who was admitted to the Emergency Department due to noticeable deficits in alertness and responsiveness after recreational ingestion of two cannabis cookies labeled to contain 40 mg “CC9” and a bite of a gummy with unknown contents. The patient experienced vomiting and visual problems, and suffered from nine days of cognitive and physical impairment. HHC-C9, a novel SSC, was detected in blood through forensic toxicological analysis. **Conclusions**: Recreational use of HHC-C9 can cause vomiting, visual disturbances, and drowsiness, potentially requiring hospital treatment. Potency, clinical effects, and toxicity of SSCs can vary significantly, and in combination with easy accessibility, SSCs pose a potential risk of intoxication to unaware consumers.

## 1. Introduction and Clinical Significance

**Figure 1:**
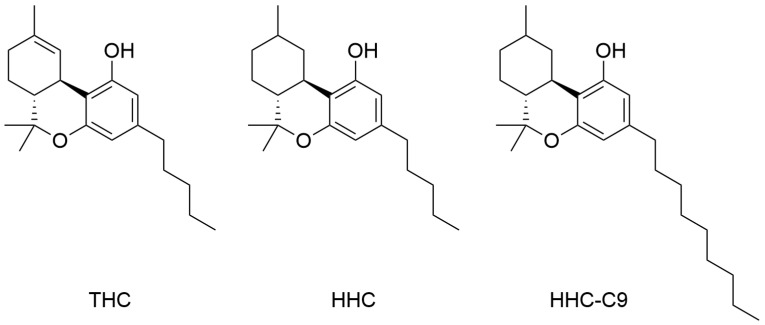
Chemical structures of (-)-*trans*-Δ^9^-tetrahydrocannabinol (THC), the primary psychoactive compound in cannabis, hexahydrocannabinol (HHC) and hexahydrocannabinonyl (HHC-C9).

Semisynthetic cannabinoids (SSCs) are a rapidly evolving group of new psychoactive substances (NPS). SSCs are structurally closely related to phytocannabinoids such as tetrahydrocannabinol (THC, Figure 1) and cannabidiol (CBD), but contain simple chemical modifications designed to retain the psychoactive properties of THC but circumvent narcotic legislation. Cannabinoids that have been detected in recent years and classified as SSCs include hexahydrocannabinol (HHC, Figure 1), tetrahydrocannabiphorol (THCP), hexahydrocannabiphorol (HHCP), tetrahydrocannabioctyl (THC-C8), and hexahydrocannabioctyl (HHC-C8). SSCs are distinct from fully synthetic cannabinoids that are not structurally related to phytocannabinoids. To date, more than 200 synthetic cannabinoids on the illegal market have been reported by the European Union Drug Agency (EUDA) [1]. SSCs are a more recent phenomenon than fully synthetic cannabinoids and are currently fewer in number, but their prevalence is increasing [2].

According to EUDA, 20 new cannabinoids were identified in Europe in 2024 alone [2]. Eighteen (90%) of these were SSCs, representing more than 40% of all new substances reported to the EU Early Warning System in 2024 [2]. Earlier, SSCs were imported from the United States to European countries, but now production has been reported to take place within Europe [2].

SSCs are easily accessible, such as through online distributors or local convenience stores, and are often promoted as legal alternatives to THC, which remains prohibited in many countries. Products containing SSCs are sold in many different forms and variations, often as herbal mixtures (spice) primarily for smoking, e-cigarette liquids, edibles (foods and candies), resins, and even papers [3,4,5]. To avoid legislative regulations, products containing SSCs are sold as “souvenirs” or “potpourri”, often marked with labels such as “Not for human consumption” or “Collector’s item”, even though they are clearly sold for recreational purposes [6]. The products are often mislabeled, and the content varies in strength and quantity between batches, which, in combination with differences in individual susceptibility, makes the effects after consumption even more unpredictable.

This case report illustrates a severe course of vomiting, visual problems, and long-lasting cognitive and physical impairment after exposure to a novel SSC, hexahydrocannabinonyl (HHC-C9, Figure 1). The exposure was analytically confirmed in a blood sample. This case provides clinically relevant information on this poorly described SSC.

## 2. Case Presentation

In May of 2025, a healthy man in his early forties consumed two cannabis cookies and a bite of a gummy in addition to a substantial amount of alcohol at a party. The first day after the ingestion, he suffered from vomiting and presented with cognitive impairment and reduced physical capacity. The cookies labeled to contain 40 mg “CC9” were bought on the internet by a friend. Two days after ingestion, the patient’s relatives contacted the Danish Poison Information Centre (DPIC) due to concerns of noticeable deficits in alertness and responsiveness. They described the patient as appearing distant, impaired, disoriented in time, and walking with a broad-based gait. They felt it was unsafe to leave him unsupervised.

DPIC advised hospital admission for observation and evaluation of potential organ dysfunction, and the patient was admitted to the Emergency Department at a regional hospital. He was persistently affected with marked latency in speech response, mild disorientation, and impaired balance. He reported visual disturbances characterized by blurred contours.

At admission, physical examination was unremarkable, including a basic neurological assessment, normal vital signs, and electrocardiography (ECG). The patient was deeply somnolent, and on arousal attempts, his speech was slurred; however, when fully aroused, he was able to speak clearly and coherently, but with a latency in verbal response. Blood tests, including renal, liver, hematology, and infection markers, were in normal range except for mild leukocytosis. Ethanol in plasma was negative. Arterial blood gas values were unremarkable. A urine immunological drug screen (NanoSticka^®^ 200-37 Panel 12, Ferle ApS, Hellebæk, Denmark) was performed and was negative for cocaine, amphetamine, methamphetamine, THC, methadone, morphine, benzodiazepines, buprenorphine, oxycodone, tramadol, fentanyl, and ketamine. Blood and urine samples were collected and sent for forensic toxicological analysis. Following 6 h of observation, the patient demonstrated increased alertness, although persistent somnolence and delayed response latency were noted. The patient was observed in the emergency department for approximately 12 h. At discharge, cerebral improvement was noted with a Glasgow Coma Score of 15, but with persistent latency in verbal response. According to the patient, subjective clinical improvement was noted on day seven, with complete recovery and return to premorbid functioning achieved by day nine.

### 2.1. Toxicological Analysis

A blood and urine sample were secured within 12 h after admission. There were no remains of the reportedly ingested cookies, but the exact name and brand of the cookies were provided by the patient. A package of cookies was purchased online 14 days after hospital admission for the purpose of forensic analysis. The purchased cookies were of the same product name and from the same manufacturer as those ingested by the patient. The brand and name of the gummy were not specified, and hence, no analysis of this product was possible.

#### 2.1.1. Blood and Urine Sample

**Figure A1:**
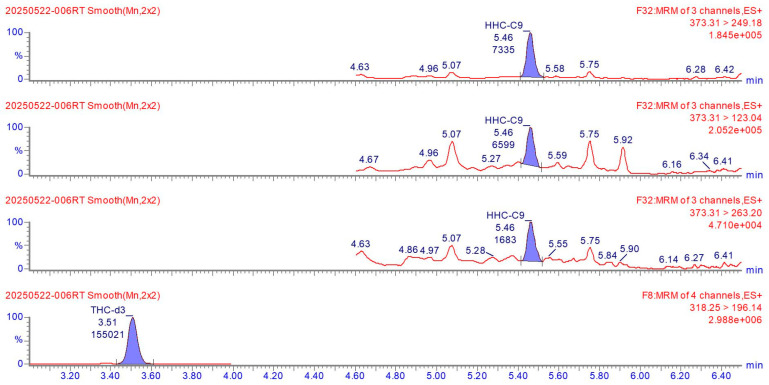
Chromatographic peak of HHC-C9 from liquid chromatography-mass spectrometry analysis of a blood sample taken three days after ingestion of cannabis cookies. Three mass transitions were recorded. The internal standard tetrahydrocannabinol-d3 is shown in the bottom chromatograms.

**Figure A2:**
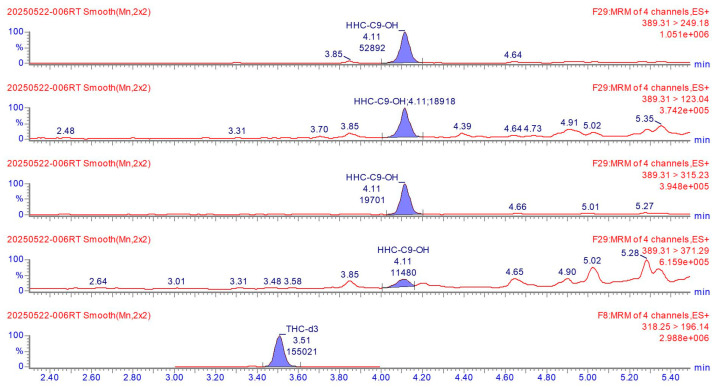
Chromatographic peak of putative HHC-C9 metabolite 11-OH-HHC-C9 from liquid chromatography-mass spectrometry analysis of a blood sample taken three days after ingestion of cannabis cookies. Four mass transitions were recorded. The internal standard tetrahydrocannabinol-d3 is shown in the bottom chromatogram.

**Figure A3:**
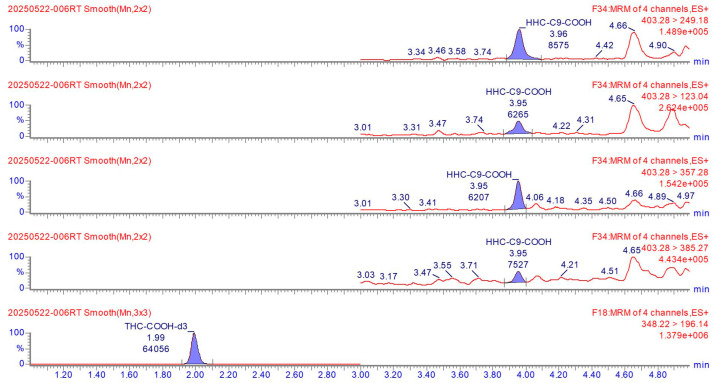
Chromatographic peak of putative HHC-C9 metabolite 11-nor-9-carboxy-HHC-C9 from liquid chromatography-mass spectrometry analysis of a blood sample taken three days after ingestion of cannabis cookies. Four mass transitions were recorded. The internal standard 11-nor-9-carboxy-tetrahydrocannabinol-d3 is shown in the bottom chromatogram.

**Figure A4:**
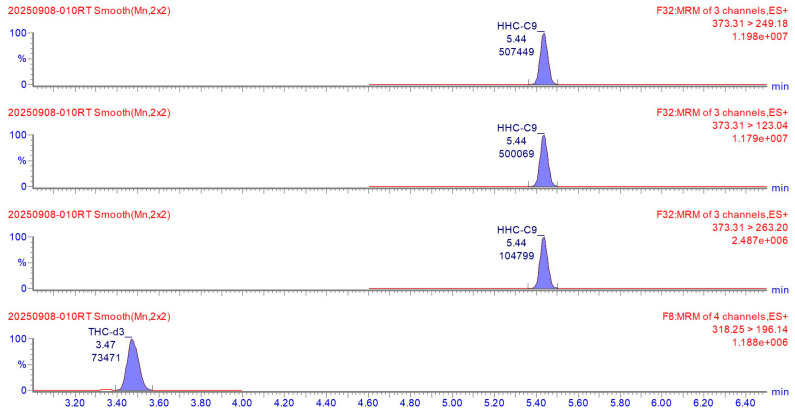
Chromatographic peak of HHC-C9 from liquid chromatography-mass spectrometry analysis of a standard solution. Three mass transitions were recorded. The internal standard tetrahydrocannabinol-d3 is shown in the bottom chromatograms.

A routine broad toxicological screening based on liquid chromatography-mass spectrometry (LC-MS) was performed on the blood and urine samples. No therapeutic drugs, drugs of abuse or toxins were initially detected. An extended screening method for SSCs was therefore employed, which revealed a low amount of HHC-C9 in the blood sample (Figure A1). Chromatographic peaks consistent with two putative metabolites of HHC-C9, 11-hydroxy-HHC-C9 and 11-nor-9-carboxy-HHC-C9, were also observed in the blood sample (Figure A2 and Figure A3). HHC-C9 and its putative metabolites were not detected in the urine sample. Retention time and ion ratios for HHC-C9 were confirmed by injection of a reference solution (Figure A4). There was no reference material for the metabolites available commercially at the time of analysis, so identification was based on predicted mass transitions and retention times. The analytical method is described in Appendix A.

#### 2.1.2. Cookies

The purchased package of cookies contained two American cookies and stated a content of 40 mg “CC9” per package. The package further stated that the “product can only be sold as a souvenir”. The cookies were subjected to a broad toxicological screening based on LC-MS as well as gas chromatography-mass spectrometry (GC-MS). The analytical methods were described previously [7]. They were found to contain HHC as the main component, with only trace amounts of HHCP and HHC-C9.

## 3. Discussion

This case report describes a case of intoxication with HHC-C9 confirmed by the detection of the compound in blood three days after ingestion. To our knowledge, there is only a single scientific paper on HHC-C9, describing the detection of the compound in a gummy candy and an electronic cigarette [8]. There is no scientific literature on the clinical effects or toxicology of the compound.

HHC is closely related to THC but has a saturated cyclohexene ring. HHC has been detected in trace amounts in the cannabis plant, but it has been sold as an SSC since 2021 [4]. HHC-C9 is an SSC similar to HHC, but where the *n*-pentyl side chain has been replaced by an *n*-nonyl chain. In vitro receptor studies have found that elongation of the side chain results in increased cannabinoid receptor 1 (CB1) binding affinity and activation [9,10]. HHC-C9 was first reported in Europe in 2025 and is among the 24 SSCs identified in the European market [2].

Case reports have documented severe intoxications after exposure to new SSCs like the closely related HHC-C8 and HHCP. Reported symptoms include prolonged sedation and unconsciousness, agitation, seizures, and confusion, underscoring the potential for increased toxicity of these compounds compared to traditional cannabis [11,12,13,14,15,16]. The clinical effects of HHC-C9 are only sparsely described. In a case from Italy, a young man was admitted to the hospital the day after smoking and ingestion of HHC-C9 [17]. Interestingly, he reported intake of products containing synthetic “CC9”. He was described as agitated and restless, and presented with conjunctival hyperemia and tremor. Treatment was symptomatic with benzodiazepines and paracetamol, and he was discharged from the hospital in good condition after 2 days. In April 2025, the Forensic Laboratory under the Department of Chemistry, University of Malta, published a Public Awareness Drug Alert due to concerns about the sale of products containing HHC-C9 [18]. The alert was issued in response to an outbreak in which seven individuals required hospital admission after HHC-C9 exposure. According to The Times of Malta, “the MAM (Medical Association of Malta) reported that their patients suffered from extreme drowsiness lasting over 24 h, vomiting, vision problems, and liver damage” [19]. These findings align with the clinical presentation of the patient described in this case, except for hepatic involvement. In the Italian case, the reported effects reflect the opposite pattern, such as agitation and restlessness.

In the present case, nothing remained of the ingested cookies for analysis. In the purchased similar cookies, the main component was determined to be HHC with only trace amounts of HHCP and HHC-C9. The trace amounts of HHCP and HHC-C9 were likely from impurities in the precursor chemicals used in the synthesis and not added deliberately. It should be noted that HHC and HHCP or their major metabolites were not detected in blood and urine samples from the patient. As the cookies were labeled to contain “CC9” and HHC-C9 was detected in the blood sample, it is likely that the batch of cookies ingested by the patient did contain HHC-C9 as the active ingredient and that the manufacturer changed the active compound in the cookies. This makes the clinical effects upon intake of cannabis products even more unpredictable and increases the risk of severe intoxication. Although exposure to HHC-C9 was likely from the ingested cookies, it cannot be completely ruled out based on the present data that the exposure was from a different product, such as the ingested gummy candy.

The widespread availability, benign or even attractive packaging, and frequent inclusion of SSCs in products such as sweets and baked goods make them particularly appealing to adolescents and even children.

## 4. Conclusions

This case describes an intoxication with HHC-C9, primarily characterized by long-lasting drowsiness, cognitive, and physical effects in addition to visual disturbances. Pronounced somnolence, sedation, and seizures have previously been reported with intoxications to the chemically closely related HHC-C8, and other related compounds such as HHCP, THCP, and THC-C8.

The rapid and uncontrolled development in the field of SSCs, the easy accessibility, varying concentration, and hence dose, as well as the potential serious health risks associated with their use point to the importance of more research and the need for monitoring these substances through toxicological analyses in biological samples as well as in cannabis products to fully understand the effects and risks of SSCs.

## Data Availability

The original contributions presented in this case report are included in this article. Further inquiries can be directed to the corresponding authors.

## Abbreviations

The following abbreviations are used in this manuscript:

DPIC	Danish Poison Information Centre
CC9	Hexahydrocannabinonyl
ECG	Electrocardiography
EUDA	European Union Drugs Agency
HHC	Hexahydrocannabinol
HHC-C8	Hexahydrocannabioctyl
HHC-C9	Hexahydrocannabinonyl
HHCP	Hexahydrocannabiphorol
THCP	Tetrahydrocannabiphorol
THC-C8	Tetrahydrocannabioctyl
NPS	New Psychoactive Substances

## Appendix A

### Analytical Method

Blood and deconjugated urine samples were prepared using solid-phase extraction. Extracted samples were analyzed on an ACQUITY UPLC I-class system with detection on Xevo TQ-S tandem mass spectrometers (Waters, Milford, MA, USA) using positive electrospray ionization. Chromatographic separation was achieved on a Kinetex EVO C18 2.6 µm column (100 mm × 2.1 mm) (Phenomenex, Torrance, CA, USA). Mobile phase A consisted of 1 mM ammonium formate and 0.1% formic acid, while mobile phase B consisted of 1:1 acetonitrile:methanol with 0.1% formic acid. Gradient elution was used, starting at 65% B, to 80% B over 3.75 min, to 100% B over 1.5 min, kept at 100% B for 0.85 min, and finally re-equilibrated at 65% B for 0.4 min. Injection volume was 10 µL. Monitored mass transitions for HHC-C9 were 373.31 > 249.18 (collision energy (c.e.) 20 eV), 373.31 > 123.04 (c.e. 35 eV), and 373.31 > 263.20 (c.e. 20 eV). Monitored mass transitions for 11-OH-HHC-C9 were 389.31 > 249.18 (c.e. 20 eV), 389.31 > 123.04 (c.e. 35 eV), 389.31 > 315.23 (c.e. 20 eV), and 389.31 > 371.29 (c.e. 15 eV). Monitored mass transitions for 11-nor-9-carboxy-HHC-C9 were 403.28 > 249.18 (c.e. 20 eV), 403.28 > 123.04 (c.e. 35 eV), 403.28 > 357.28 (c.e. 20 eV), and 403.28 > 385.27 (c.e. 15 eV). (6a*R*,9*R*,10a*R*)-hexahydrocannabinonyl was from Cayman Chemical (Ann Arbor, MI, USA).
